# 

**Published:** 1876-11

**Authors:** 


Vol. XXXIII.
Number 11.

November, 1876.

NEW SERIES

Vol. 1.

Number 11.

THE

### CHICAGO MEDICAL Journal and Examiner

PUBLISHED MONTHLY.

**EDITOR:**
WILLIAM H. BYFORD, A.M., M.D.

**ASSOCIATE EDITORS:**
JAMES H. ETHERIDGE, M.D.
NORMAN BRIDGE, M.D.
JAS. NEVINS HYDE, A.M., M.D.
FERD. C. HOTZ, M.D.

Published under the auspices of the

*CHICAGO MEDICAL PRESS ASSOCIATION.*

**TERMS—FOUR DOLLARS PER ANNUM, IN ADVANCE.**
**POSTAGE FREE.**

FOLIA SUA AD SANITATEM GENTIUM

S.G.O. INDEXED

CHICAGO:
W. B. KEEN, COOKE & CO., Publishers,
Nos. 113 and 115 State Street.

☞ Our Patrons and Contributors will kindly assist The Chicago

